# The reliability of a new method for inter-metatarsal angle assessment in hallux valgus surgery

**DOI:** 10.1186/1757-1146-4-S1-O1

**Published:** 2011-05-20

**Authors:** Simon Adam

**Affiliations:** 1 NHS Lambeth, Department of Podiatric Surgery, London, UK

## Background

The assessment of the intermetatarsal angle associated with hallux valgus has been investigated numerous times within the literature with multiple assessment methods, often producing conflicting results, being presented. The centre of the head method as described by Mitchell in 1958 is a common assessment method however the potential complications associated with post medial eminence cheilectomy is of concern.

## Methods

The two presented methods including the standard method of measurements using the centre of the metatarsal head’s articular surface was assessed and directly compared to the newly presented ‘lateral’ method whom use the most lateral border of the metatarsal head. 33 patients with pre and post operative AP/DP radiographic images were assessed by three clinicians of varied experience on three separate occasions using the two assessment methods. Pearson's Correlation Coefficient (PCC) and Bland Altman plots were used to assess agreement between the methods. Whilst ANOVA and Intrarclass Correlation Coefficient were used to assess interobserver and intraobserver reliability.

## Results

The correlation between the two assessment methods was strong with PCC demonstrating 0.903 on pre and postoperative assessment together. The agreement between the two methods showed no statistically significant difference (p=0.392) on the preoperative assessment results obtained. Postoperatively there was a statistically significant difference (p<0.001) with the Mitchell method demonstrating a +2.8° increase in IMA correction post intervention compared to the lateral method. The ICC demonstrated both methods to have very high intraobserver (range, 0.821 to 0.935) values although the lateral method was more superior in four of the six assessments undertaken. Both methods demonstrated very high interobserver (range 0.872 to 0.944) values with the lateral method demonstrating more superior results in both pre and postoperative assessments.

## Conclusions

Intermetatarsal angles made on pre and postoperative radiographs were more reliable when using the newly presented lateral method. Further investigation is required to identify why the Mitchell method, on average, produces a 3° improved Intermetatarsal angle post intervention. Based on the results presented the lateral method should be implemented into the clinical setting.

